# How to model temporal changes in comorbidity for cancer patients using prospective cohort data

**DOI:** 10.1186/s12911-015-0217-8

**Published:** 2015-11-18

**Authors:** Lars Lindhagen, Mieke Van Hemelrijck, David Robinson, Pär Stattin, Hans Garmo

**Affiliations:** Uppsala Clinical Research Center, Uppsala, Sweden; Division of Cancer Studies, Cancer Epidemiology Group, Research Oncology, King’s College London, School of Medicine, 3rd Floor, Bermondsey Wing, Guy’s Hospital, London, SE1 9RT UK; Karolinska Institute, Institue of Environmental Medicine, Stockholm, Sweden; Department of Urology, Ryhov County Hospital, Jonkoping, Sweden; Department of Surgical and Perioperative Sciences, Urology and Andrology, Umeå University, Umeå, Sweden; Regional Cancer Centre, Uppsala Örebro, Uppsala, Sweden

**Keywords:** Temporal changes, Comorbidity, Cancer, Cox proportional hazards, State transition model

## Abstract

**Background:**

The presence of comorbid conditions is strongly related to survival and also affects treatment choices in cancer patients. This comorbidity is often quantified by the Charlson Comorbidity Index (CCI) using specific weights (1, 2, 3, or 6) for different comorbidities. It has been shown that the CCI increases at different times and with different sizes, so that traditional time to event analysis is not adequate to assess these temporal changes. Here, we present a method to model temporal changes in CCI in cancer patients using data from PCBaSe Sweden, a nation-wide population-based prospective cohort of men diagnosed with prostate cancer. Our proposed model is based on the assumption that a change in comorbidity, as quantified by the CCI, is an irreversible one-way process, i.e., CCI accumulates over time and cannot decrease.

**Methods:**

CCI was calculated based on 17 disease categories, which were defined using ICD-codes for discharge diagnoses in the National Patient Register. A state transition model in discrete time steps (i.e., four weeks) was applied to capture all changes in CCI. The transition probabilities were estimated from three modelling steps: 1) Logistic regression model for vital status, 2) Logistic regression model to define any changes in CCI, and 3) Poisson regression model to determine the size of CCI change, with an additional logistic regression model for CCI changes ≥ 6. The four models combined yielded parameter estimates to calculate changes in CCI with their confidence intervals.

**Results:**

These methods were applied to men with low-risk prostate cancer who received active surveillance (AS), radical prostatectomy (RP), or curative radiotherapy (RT) as primary treatment. There were large differences in CCI changes according to treatment.

**Conclusions:**

Our method to model temporal changes in CCI efficiently captures changes in comorbidity over time with a small number of regression analyses to perform – which would be impossible with tradition time to event analyses. However, our approach involves a simulation step that is not yet included in standard statistical software packages. In our prostate cancer example we showed that there are large differences in development of comorbidities among men receiving different treatments for prostate cancer.

**Electronic supplementary material:**

The online version of this article (doi:10.1186/s12911-015-0217-8) contains supplementary material, which is available to authorized users.

## Background

Comorbidities are known to affect cancer survival, with 5-year mortality hazard ratios ranging from 1.1 to 5.8 depending on levels of comorbidity [1]. A recent systematic review reported that presence of specific severe comorbidities or psychiatric disorders was associated with delayed cancer diagnosis, whereas men and women with chronic diseases with regular medical consultations had their cancer detected at an earlier stage. Furthermore, a smaller proportion of cancer patients with comorbidities received standard treatment than patients without comorbidities, and in addition their chance of completing a course of cancer treatment was lower [1]. For instance, a study by Griffiths et al. examined impact of comorbidity 12 months before to 84 months after a breast cancer diagnosis. Overall, 10 % of the women had undetected comorbidity prior to diagnosis, and these women received less aggressive treatment and had higher all-cause mortality than otherwise healthy women [2]. In the case of prostate cancer, it has been found that prostate cancer mortality at ten years after diagnosis was similar for men aged 70 with high-risk disease, regardless of their comorbidities: 15 % in men with no other comorbidities and 14 % in men with moderate levels of comorbidities. In contrast, all-cause mortality was substantially lower for men with no comorbidities (34 %), than for men with moderate levels of comorbidity (47 %) [3].

Thus, changes in comorbidity are important to include into analysis as it affects treatment choice and disease progression of cancer patients. Changes in comorbidity, as measured by the Charlson Comorbidity index (CCI) [4, 5], occur multiple times and with different sizes (1, 2, 3, or 6), which is difficult to describe using traditional time to event analysis. Therefore, we present a method using a multi-state approach to model temporal changes in comorbidity for cancer patients using data from PCBaSe Sweden, a national-wide population-based prospective cohort of men diagnosed with PCa between 1992 and 2012. The proposed method is based on the assumption that a CCI change is an irreversible process, i.e., CCI accumulates over time and cannot decrease.

## Methods

### Study population and data collection

Prostate Cancer data Base Sweden (PCBaSe Sweden) consists of the National Prostate Cancer Register of Sweden (NPCR) linked to a number of different nationwide registers [6]. NPCR became nationwide in 1998 and covers 98 % of all newly diagnosed, biopsy-confirmed cases of prostate cancer, as compared to the Swedish Cancer Registry. Information in PCBaSe on age, serum PSA, primary treatment, tumour grade and stage, and cause and date of death was used. Prostate cancer risk category was defined according to a modification of the National Comprehensive Cancer Network Guideline [7]. The linkage of PCBaSe was approved by the Research Ethics Board at Umeå University.

For the current analysis, we selected men recorded in NPCR with low risk prostate cancer, diagnosed between 2003 and 2012, who received active surveillance (AS), radical prostatectomy (RP), or curative radiotherapy (RT) as primary treatment. Comorbidity was measured with the CCI and was retrieved from the National Patient Register and the National Cancer Register [4, 5]. We used 17 groups of diseases with specific weights (1, 2, 3, or 6) assigned to each disease category, as defined by Charlson et al. [8]. Information on these diseases was based on ICD (International Classification of Diseases) codes for discharge diagnoses. The actual day for each event was retrieved from the national healthcare registers. We then applied the specified weights to the 17 different types of events to calculate the CCI on a daily basis. Thus, CCI is a time-dependent covariate that could change multiple times during follow-up. An overview of all covariates used in our analyses is provided in Additional file 1: Tables S1 and S2.

Data cannot is not freely available following the legislation of the Swedish Public Access to Information and Secrecy Act. However, data can be made available to researchers upon request. The steering groups of NPCR and PCBaSe welcome external collaborations. For more information please see www.npcr.se/in-english where registration forms, manuals, and annual reports from NPCR are found as well as a full list of publications from PCBaSe.

### Analysis

**Fig. 1:**
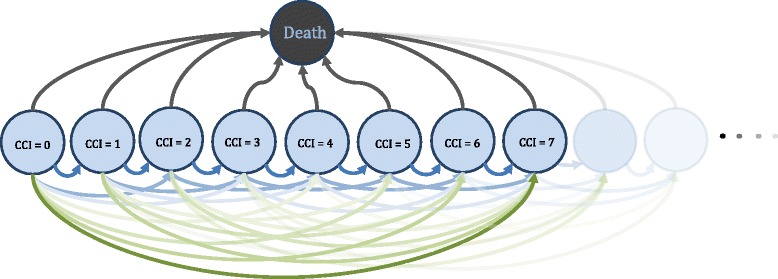
Possible CCI-states (*blue*) and final death-state (*black*) in the state transition model. The arrows reflect the possible changes in CCI and the possibility of death

We propose a method based on a state transition model approach with states and state transitions, as illustrated in Fig. 1. CCI changes are considered irreversible, i.e., CCI accumulates over time and cannot decrease as indicated by the arrows only pointing towards higher CCI states in Fig. 1. In each CCI-state there is a possibility of death, indicated by the arrows pointing towards the death state. Due to the large number of states and transitions, the proposed model was simplified as described below. An overview of the R-codes used for these models is provided in Additional file 1: Table S3.

Firstly, we simplified follow-up time by discretizing in time steps whereby an individual could experience death, a change in CCI of any size, or remain in the previous CCI state at each time step. In our prostate cancer example, we chose a time step of four weeks. The discretised data was arranged using long format, i.e., each study subject was represented by several rows of data, one for each time step in which the study subject was still alive. Age and CCI were updated at each time step.

**Fig. 2:**
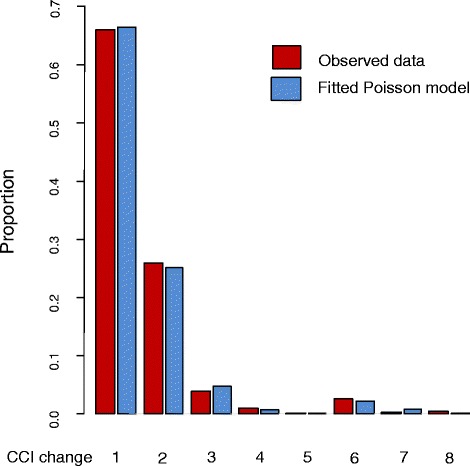
Observed and modelled changes in CCI

Next, we estimated the state transition probabilities in three-step process:

- Step 1: We started by determining a person’s vital status at the end of each time step. The probability of death was modelled with a logistic regression analysis applied to the long format dataset. This first model used death (yes/no) as the outcome and age (linear), CCI (linear), and their statistical interaction as regressors.
- Step 2: When a man was alive at the end of a time step, we determined whether a change in CCI had occurred. This was modelled with a logistic regression model using CCI-change (yes/no) as the outcome. The regressors in this second model were treatment (AS/RP/RT), age (linear), CCI (0/1/2/3/4+), time since previous CCI change (1, 2–3, 4–6, or >6 months, with the latter also including no previous CCI change), time since RP (1, 2–3, 4–6, or >6 months, with the latter also including no RP), time since RT (1, 2–3, 4–6, or >6 months, with the latter also including no RT), and a statistical interaction between age and treatment.
- Step 3: When a change in CCI occurred, the final step defined the size of this CCI change. Here, we made a second simplification based on the observation that changes in CCI size approximately follow a Poisson distribution, with the exception for changes ≥ 6 (Fig. 2). This exception reflects diseases that contribute to CCI with a weight of 6. Therefore, this final third step of the transition model was split into two parts:
  - First, we applied Poisson regression to the subset of the long dataset where CCI changes occurred. Transformed CCI change was the outcome and calculated as follows: All changes were decreased by 1, except changes of size ≥6, which were decreased by 6. Thus, the smallest possible outcome was zero, corresponding to a CCI-change of 1 or 6. The same regressors as in the model of Step 2 were used.
  - To handle CCI changes ≥6, an additional logistic regression model was applied with CCI change ≥” (yes/no) as the outcome. In this model, we used the following regressors: treatment (AS/RP/RT), CCI (0/1/2+ together with a linear term), time since previous CCI change (as in Step 2), and age (linear).

The above steps yielded a set of parameter estimates which were used to simulate CCI in a microsimulation [9], i.e., a simulation of CCI changes in individual study subjects. In this simulation, outcomes according to models 1, 2, 3a, and 3b were generated. The dichotomous outcome from Step 3b indicated whether the simulated Poisson outcome from Step 3a should be increased by 1 or 6 to recover the actual CCI-change. We performed this simulation of CCI development in 1,000,000 study subjects with pre-defined values for primary treatment, initial CCI, and age.

The modelling-simulation approach [9] made it possible to calculate confidence intervals for the predicted CCI at specific time points, and in particular differences in CCI between exposure groups. The confidence intervals were computed using the unscented transform [10]. This method resembles bootstrap techniques in that the final estimate varies as a result of repeated simulations using different values of the regression coefficients. The different estimates were then used to calculate the confidence intervals. However, the unscented transform is more efficient since the number of simulations needed is limited to about twice the number of regression coefficients, a much smaller number when compared to traditional bootstrap techniques. In accordance with the unscented transform, the combinations of regression coefficients were chosen deterministically, based on the estimated covariance matrices.

## Results and discussion

Table 1 illustrates that baseline characteristics in the three treatment groups were substantially different. For instance, men treated with RP were younger than men on AS or RT. Prostate-specific antigen (PSA) levels were higher for men on RT than for men on AS or RP. It is therefore of interest to identify how the effect of treatment on changes in CCI was affected by age and other predictors.

**Table 1:** Baseline characteristics of the study population in Prostate Cancer data Base Sweden (PCBaSe) 3.0

	Active surveillance (*n* = 7 544)	Radical prostatectomy (*n* = 9 959)	Curative radio therapy (*n* = 2 734)	Total (*n* = 20,237)
Age, mean (sd)	65.4 (6.0)	61.7 (5.9)	64.8 (5.7)	63.5 (6.2)
Age, *n* (%)				
≤55	362 (4.8)	1315 (13.2)	147 (5.4)	1824 (9.0)
56–60	997 (13.2)	2280 (22.9)	435 (15.9)	3712 (18.3)
61–65	2042 (27.1)	3314 (33.3)	781 (28.6)	6137 (30.3)
66–70	2475 (32.8)	2433 (24.4)	846 (30.9)	5754 (28.4)
70+	1668 (22.1)	617 (6.2)	525 (19.2)	2810 (13.9)
Educational level, *n* (%)				
High	2116 (28.0)	3327 (33.4)	731 (26.7)	6174 (30.5)
Low	2198 (29.1)	2485 (25.0)	841 (30.8)	5524 (27.3)
Middle	3206 (42.5)	4109 (41.3)	1147 (42.0)	8462 (41.8)
Missing	24 (0.3)	38 (0.4)	15 (0.5)	77 (0.4)
CCI at PCa diagnosis, *n* (%)				
0	6288 (83.4)	9008 (90.5)	2276 (83.2)	17,572 (86.8)
1	734 (9.7)	596 (6.0)	295 (10.8)	1625 (8.0)
2	371 (4.9)	268 (2.7)	108 (4.0)	747 (3.7)
3+	151 (2.0)	87 (0.9)	55 (2.0)	293 (1.4)
T-stage, *n* (%)				
T1a	513 (6.8)	106 (1.1)	17 (0.6)	636 (3.1)
T1b	126 (1.7)	61 (0.6)	22 (0.8)	209 (1.0)
T1c	5807 (77.0)	7152 (71.8)	1796 (65.7)	14,755 (72.9)
T2	1078 (14.3)	2621 (26.3)	887 (32.4)	4586 (22.7)
TX/Missing	20 (0.3)	19 (0.2)	12 (0.4)	51 (0.3)
N-stage, *n* (%)				
N0	443 (5.9)	980 (9.8)	146 (5.3)	1569 (7.8)
NX	7101 (94.1)	8979 (90.2)	2588 (94.7)	18,668 (92.2)
PSA, mean (sd)	5.5 (2.0)	5.8 (1.9)	6.1 (1.9)	5.7 (2.0)
PSA, *n* (%)				
0–2.0	277 (3.7)	157 (1.6)	28 (1.0)	462 (2.3)
2.1–4.0	1578 (20.9)	1874 (18.8)	368 (13.5)	3820 (18.9)
4.1–6.0	2868 (38.0)	3910 (39.3)	991 (36.2)	7769 (38.4)
6.1–8.0	1848 (24.5)	2568 (25.8)	838 (30.7)	5254 (26.0)
8.1–10	973 (12.9)	1450 (14.6)	509 (18.6)	2932 (14.5)

**Fig. 3:**
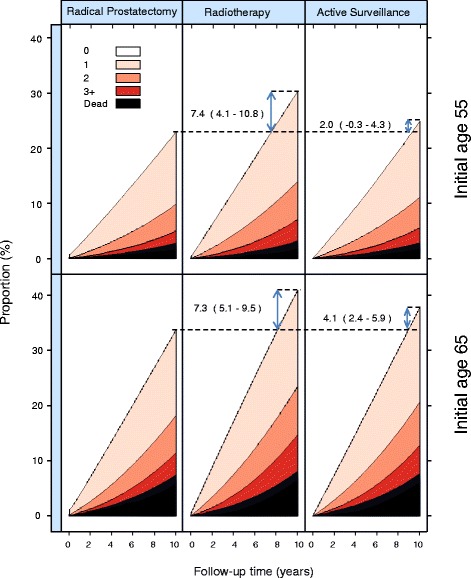
CCI levels by time of follow-up for men aged 55 or 65 with CCI = 0 at time of diagnosis, who underwent active surveillance, radical prostatectomy, or radiotherapy. CCI levels were modelled with a state transition model. Absolute differences (and 95 % Confidence Intervals) in the proportion of men remaining on CCI = 0 by treatment groups were also estimated (as indicated by the blue arrows)

Figure 3 represents the changes in CCI during 10 years following AS, RP, and RT for men with prostate cancer aged 55 and 65, with initial CCI = 0. It also shows absolute differences (with 95 % confidence intervals) in the proportion of men remaining on CCI = 0 by treatment regime (RT/AS and RP). Follow-up time started at treatment initiation. There was a rapid increase in CCI for men treated with RP or RT. This is likely explained by the fact that in Sweden all medical conditions of a patient are included in the discharge diagnoses following an in-hospital episode. Therefore, conditions that were previously not recorded will occur for the first time after hospitalisation despite the fact the patient may have had this condition for a long time. Furthermore, there was a more rapid increase in CCI for men treated with RT than AS. For instance, at 10 years after treatment a higher proportion of men age 65 had died or changed CCI after RT than after RP, 7.3 % (95 % CI: 5.1–9.5). The corresponding value for AS versus RP was 4.1 % (95 % CI: 2.4–5.9).

The following section further clarifies the model specifications and simplifications used in our approach to model temporal changes in comorbidity. Our choice of four-week time steps was a trade-off between precision of dates when CCI changes occurred and computer resources in terms of memory and CPU. A smaller time step would make the long format dataset very large. The implications of our choice of time steps can also be evaluated by looking at time intervals with several CCI changes. For instance, our time steps of four weeks resulted in 2 % of the CCI changes (updated on a daily basis) being pooled with other changes in the same time interval (data not shown). A smaller time step would have alleviated this problem, but at the expense of increased computer load. Moreover, larger time steps would also require a thorough examination of the assumption that changes in CCI follow a Poisson distribution.

It is also important to note that the Poisson distribution, as observed for the size of CCI changes in our example, was purely based on investigation of our data and not justified by any probability theory arguments. If all the CCI changes would have been of size one, a theoretical motivation for the Poisson distribution would have been justifiable. Nevertheless, the weights used to calculate the CCI (1, 2, 3, or 6) make the theoretical basis for usage of the Poisson-distribution limited. Thus, this simplification may need further investigation in other research situations as it is not a given that a Poisson distribution will fit any type of data and, as suggested above, this may also depend on the size of the time steps.

**Fig. 4:**
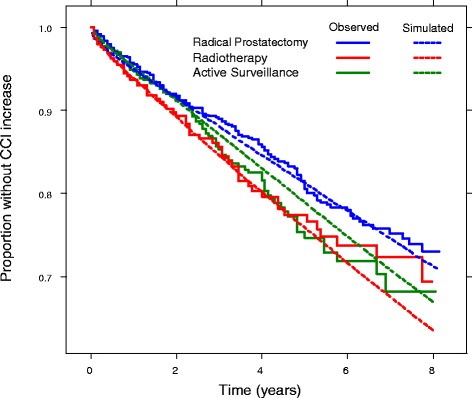
Modelled and observed time to first increase in CCI of ≥1 units for men with prostate cancer aged 65 years with CCI = 1 at time of diagnosis

Further justification of the validity of our transition stage models follows from a comparison between simulated and observed data on CCI changes (Fig. 4). Simulated data were obtained using the approach described above, whereas observed data were taken from the traditional Kaplan-Meier estimates with the endpoint of an increase in CCI of at least one unit. Figure 4 shows a comparison for men aged 65 with CCI = 1 at time of prostate cancer diagnosis. The close agreement between the curves for observed and simulated data supports the choice of the models defined above.

Finally, our transition stage model required less model specifications than the traditional time-to-event approach. It captured the complete change in CCI over time using four regression models (three logistic and one Poisson model). This problem would have been difficult to address using traditional survival analysis such as Cox proportional hazards regression. These models only deal with time to a specific event. In the current setting such an event could, for example, be a transition from CCI = 1 to the CCI = 3. More specifically, each arrow in Fig. 1 could correspond to such an event, so that each arrow in Fig. 1 would correspond to a separate Cox model. In practice the cumulated CCI rarely exceeds 15. If we restrict the problem to CCI ≤ 15, the number of models needed to fit would be 16 + 15 + … + 1 = 136. When limiting the problem to CCI ≤ 2, still six Cox models would be needed, three for CCI and three for death. Even when using a Cox model for each size of CCI-change, several different Cox models would be required. Thus, the number of regression analyses needed when applying our proposed method is comparatively small.

Moreover, it is not well-defined how to perform simulations like the ones proposed, based on a set of Cox models. To simulate single event times from a Cox model one could use the estimated hazard function. However, when calculating confidence intervals, a measure of the uncertainty for the hazard function estimate is required. Such an estimate is possible to retrieve using the continuous-time stochastic processes described in [11]. Although possible, this problem is complicated even when CCI ≤ 2, because the variance-covariance matrix is practically infinite dimensional. In our complex simulation, where the results from several different models need to be combined, the mathematical difficulties would be overwhelming.

The simulation in our framework is much simpler since we repeatedly take a small step forward in time. This approach also makes it possible to estimate the uncertainty by using the unscented transform because the number of regression coefficients is limited and the estimated variance-covariance matrix is readily available for each time step.

## Conclusions

Our method to model temporal changes in CCI efficiently captures the changes in comorbidity over time with a small number of regression analyses to perform. However, our approach involves a simulation step that is not yet included in standard statistical software packages.

## Data availability

Data used for the current study (i.e., treatment, date of prostate cancer diagnosis, age at prostate cancer diagnosis, date of study entry, last date of follow-up, censoring, date of radical prostatectomy, date of radiotherapy, and all levels and dates of CCI changes) can be retrieved by contacting hans.garmo@kcl.ac.uk.

The steering groups of NPCR and PCBaSe welcome external collaborations. For more information please see www.npcr.se/in-english where registration forms, manuals, and annual reports from NPCR are found as well as a full list of publications from PCBaSe.

## Additional file

Additional file 1: Table S1. Variables used in the long dataset. **Table S2.** Subset of data including three study subjects transformed to long format. **Table S3.** R-codes used for subset selection and models 1, 2, 3a, and 3b. (DOCX 46 kb)

## Abbreviations

95 % CI: 95 % Confidence interval
AS: Active surveillance
CCI: Charlson comorbidity index
PCa: Prostate cancer
RP: Radical prostatectomy
RT: Radiotherapy
