# Supplementary material for: How to model temporal changes in comorbidity for cancer patients using prospective cohort data
Source: BMC Med Inform Decis Mak. 2015 Nov 18;15:96. doi: 10.1186/s12911-015-0217-8 (PMC4652373; doi:10.1186/s12911-015-0217-8)
Supplement: Additional file 1: Table S1. — Variables used in the long dataset. Table S2. Subset of data including three study subjects transformed to long format. Table S3. R-codes used for subset selection and models 1, 2, 3a, and 3b. (DOCX 46 kb) [file 12911_2015_217_MOESM1_ESM.docx]

**Appendix A**

**Table A1**.Variables used in the long dataset.

| **Variable** | **Description** | **Values** |
| --- | --- | --- |
| ID | Study identification number | 1, 2, 3 …. |
| State | Present treatment | AS / RP / RT |
| Age | Age of men at start of time step | Numeric |
| TSRP | Time since Radical Prostatectomy (28 days) | Numeric |
| TSRT | Time since Radiotherapy (28 days) | Numeric |
| Dead | Vital status | True/False (T/F) |
| t0 | Time in present state | Numeric |
| CCI | CCI level at start of time step | 0, 1, 2 …. |
| CCI.change | CCI change during time step | 0, 1, 2 …. |
| TSC | Number of time steps since last CCI change | NA / 1, 2, 3 …. |
| CCI.fct | CCI factorised | 0 / 1 / 2 / 3 / 4+ |
| CCI.fct.6p | CCI factorised | 0 / 1 / 2+ |
| TSC.1 | TSC= 1 | True/False |
| TSC.23 | TSC= 2 or 3 | True/False |
| TSC.46 | TSC= 4, 5, or 6 | True/False |
| TSRP.1 | TSRP= 1 | True/False |
| TSRP.23 | TSRP= 2 or 3 | True/False |
| TSRP.46 | TSRP= 4, 5, or 6 | True/False |
| TSRT.1 | TSRT= 1 | True/False |
| TSRT.23 | TSRT= 2 or 3 | True/False |
| TSRT.46 | TSRT= 4, 5, or 6 | True/False |

**Table A2**. Subset of data including three study subjects transformed to long format.

| **ID** | **State** | **Diag.state** | **Age** | **TSRP** | **TSRT** | **Dead** | **t0** | **CCI** | **CCI.change** | **TSC** | **CCI.fct** | **CCI.fct.6p** | **TSC.1** | **TSC.23** | **TSC.46** | **TSRP.1** | **TSRP.23** | **TSRP.46** | **TSRT.1** | **TSRT.23** | **TSRT.46** |
| --- | --- | --- | --- | --- | --- | --- | --- | --- | --- | --- | --- | --- | --- | --- | --- | --- | --- | --- | --- | --- | --- |
| 1 | AS | AS | 66,24 | NA | NA | F | 0 | 2 | 0 | NA | 2 | 2+ | F | F | F | F | F | F | F | F | F |
| 1 | AS | AS | 66,32 | NA | NA | F | 1 | 2 | 0 | NA | 2 | 2+ | F | F | F | F | F | F | F | F | F |
| 1 | AS | AS | 66,39 | NA | NA | F | 2 | 2 | 2 | NA | 2 | 2+ | F | F | F | F | F | F | F | F | F |
| 1 | AS | AS | 66,47 | NA | NA | F | 3 | 4 | 1 | 1 | 4+ | 2+ | T | F | F | F | F | F | F | F | F |
| 1 | AS | AS | 66,55 | NA | NA | F | 4 | 5 | 0 | 1 | 4+ | 2+ | T | F | F | F | F | F | F | F | F |
| 1 | AS | AS | 66,62 | NA | NA | F | 5 | 5 | 0 | 2 | 4+ | 2+ | F | T | F | F | F | F | F | F | F |
| 1 | AS | AS | 66,70 | NA | NA | F | 6 | 5 | 1 | 3 | 4+ | 2+ | F | T | F | F | F | F | F | F | F |
| 1 | AS | AS | 66,78 | NA | NA | F | 7 | 6 | 0 | 1 | 4+ | 2+ | T | F | F | F | F | F | F | F | F |
| 1 | AS | AS | 66,85 | NA | NA | F | 8 | 6 | 0 | 2 | 4+ | 2+ | F | T | F | F | F | F | F | F | F |
| 1 | AS | AS | 66,93 | NA | NA | F | 9 | 6 | 0 | 3 | 4+ | 2+ | F | T | F | F | F | F | F | F | F |
| 1 | AS | AS | 67,01 | NA | NA | F | 10 | 6 | 0 | 4 | 4+ | 2+ | F | F | T | F | F | F | F | F | F |
| 1 | AS | AS | 67,08 | NA | NA | F | 11 | 6 | 0 | 5 | 4+ | 2+ | F | F | T | F | F | F | F | F | F |
| 1 | AS | AS | 67,16 | NA | NA | F | 12 | 6 | 0 | 6 | 4+ | 2+ | F | F | T | F | F | F | F | F | F |
| 1 | AS | AS | 67,24 | NA | NA | F | 13 | 6 | 0 | 7 | 4+ | 2+ | F | F | F | F | F | F | F | F | F |
| 1 | AS | AS | 67,31 | NA | NA | T | 14 | 6 | 0 | 8 | 4+ | 2+ | F | F | F | F | F | F | F | F | F |
|  |  |  |  |  |  |  |  |  |  |  |  |  |  |  |  |  |  |  |  |  |  |
| 2 | AS | AS | 76,68 | -1,36 | NA | F | 0 | 0 | 0 | NA | 0 | 0 | F | F | F | F | F | F | F | F | F |
| 2 | AS | AS | 76,76 | -1,28 | NA | F | 1 | 0 | 0 | NA | 0 | 0 | F | F | F | F | F | F | F | F | F |
| 2 | AS | AS | 76,83 | -1,21 | NA | F | 2 | 0 | 0 | NA | 0 | 0 | F | F | F | F | F | F | F | F | F |
| 2 | AS | AS | 76,91 | -1,13 | NA | F | 3 | 0 | 0 | NA | 0 | 0 | F | F | F | F | F | F | F | F | F |
| 2 | AS | AS | 76,99 | -1,05 | NA | F | 4 | 0 | 0 | NA | 0 | 0 | F | F | F | F | F | F | F | F | F |
| 2 | AS | AS | 77,06 | -0,98 | NA | F | 5 | 0 | 0 | NA | 0 | 0 | F | F | F | F | F | F | F | F | F |
| 2 | AS | AS | 77,14 | -0,90 | NA | F | 6 | 0 | 0 | NA | 0 | 0 | F | F | F | F | F | F | F | F | F |
| 2 | AS | AS | 77,22 | -0,82 | NA | F | 7 | 0 | 0 | NA | 0 | 0 | F | F | F | F | F | F | F | F | F |
| 2 | AS | AS | 77,29 | -0,75 | NA | F | 8 | 0 | 0 | NA | 0 | 0 | F | F | F | F | F | F | F | F | F |
| 2 | AS | AS | 77,37 | -0,67 | NA | F | 9 | 0 | 6 | NA | 0 | 0 | F | F | F | F | F | F | F | F | F |
| 2 | AS | AS | 77,45 | -0,59 | NA | F | 10 | 6 | 0 | 1 | 4+ | 2+ | T | F | F | F | F | F | F | F | F |
| 2 | AS | AS | 77,52 | -0,52 | NA | F | 11 | 6 | 0 | 2 | 4+ | 2+ | F | T | F | F | F | F | F | F | F |
| 2 | AS | AS | 77,60 | -0,44 | NA | F | 12 | 6 | 0 | 3 | 4+ | 2+ | F | T | F | F | F | F | F | F | F |
| 2 | AS | AS | 77,68 | -0,36 | NA | F | 13 | 6 | 0 | 4 | 4+ | 2+ | F | F | T | F | F | F | F | F | F |
| 2 | AS | AS | 77,75 | -0,29 | NA | F | 14 | 6 | 0 | 5 | 4+ | 2+ | F | F | T | F | F | F | F | F | F |
| 2 | AS | AS | 77,83 | -0,21 | NA | F | 15 | 6 | 2 | 6 | 4+ | 2+ | F | F | T | F | F | F | F | F | F |
| 2 | AS | AS | 77,91 | -0,13 | NA | F | 16 | 8 | 0 | 1 | 4+ | 2+ | T | F | F | F | F | F | F | F | F |
| 2 | AS | AS | 77,98 | 0,06 | NA | F | 17 | 8 | 0 | 2 | 4+ | 2+ | F | T | F | F | F | F | F | F | F |
| 2 | RP | AS | 78,04 | 0,00 | NA | F | 0 | 8 | 0 | 3 | 4+ | 2+ | F | T | F | T | F | F | F | F | F |
| 2 | RP | AS | 78,12 | 0,08 | NA | F | 1 | 8 | 0 | 4 | 4+ | 2+ | F | F | T | F | T | F | F | F | F |
| 2 | RP | AS | 78,19 | 0,15 | NA | F | 2 | 8 | 0 | 5 | 4+ | 2+ | F | F | T | F | T | F | F | F | F |
| 2 | RP | AS | 78,27 | 0,23 | NA | F | 3 | 8 | 0 | 6 | 4+ | 2+ | F | F | T | F | F | T | F | F | F |
| 2 | RP | AS | 78,35 | 0,31 | NA | F | 4 | 8 | 0 | 7 | 4+ | 2+ | F | F | F | F | F | T | F | F | F |
| 2 | RP | AS | 78,42 | 0,38 | NA | F | 5 | 8 | 0 | 8 | 4+ | 2+ | F | F | F | F | F | T | F | F | F |
| 2 | RP | AS | 78,50 | 0,46 | NA | F | 6 | 8 | 0 | 9 | 4+ | 2+ | F | F | F | F | F | F | F | F | F |
| 2 | RP | AS | 78,58 | 0,54 | NA | F | 7 | 8 | 0 | 10 | 4+ | 2+ | F | F | F | F | F | F | F | F | F |
| 2 | RP | AS | 78,65 | 0,61 | NA | F | 8 | 8 | 0 | 11 | 4+ | 2+ | F | F | F | F | F | F | F | F | F |
| 2 | RP | AS | 78,73 | 0,69 | NA | F | 9 | 8 | 0 | 12 | 4+ | 2+ | F | F | F | F | F | F | F | F | F |
| 2 | RP | AS | 78,81 | 0,77 | NA | F | 10 | 8 | 0 | 13 | 4+ | 2+ | F | F | F | F | F | F | F | F | F |
| 2 | RP | AS | 78,88 | 0,84 | NA | F | 11 | 8 | 0 | 14 | 4+ | 2+ | F | F | F | F | F | F | F | F | F |
| 2 | RP | AS | 78,96 | 0,92 | NA | F | 12 | 8 | 0 | 15 | 4+ | 2+ | F | F | F | F | F | F | F | F | F |
| 2 | RP | AS | 79,04 | 1,00 | NA | F | 13 | 8 | 0 | 16 | 4+ | 2+ | F | F | F | F | F | F | F | F | F |
| 2 | RP | AS | 79,11 | 1,07 | NA | F | 14 | 8 | 0 | 17 | 4+ | 2+ | F | F | F | F | F | F | F | F | F |
| 2 | RP | AS | 79,19 | 1,15 | NA | F | 15 | 8 | 0 | 18 | 4+ | 2+ | F | F | F | F | F | F | F | F | F |
| 2 | RP | AS | 79,27 | 1,23 | NA | T | 16 | 8 | 0 | 19 | 4+ | 2+ | F | F | F | F | F | F | F | F | F |
|  |  |  |  |  |  |  |  |  |  |  |  |  |  |  |  |  |  |  |  |  |  |
| 3 | AS | AS | 70,19 | -0,82 | NA | F | 0 | 0 | 0 | NA | 0 | 0 | F | F | F | F | F | F | F | F | F |
| 3 | AS | AS | 70,27 | -0,74 | NA | F | 1 | 0 | 0 | NA | 0 | 0 | F | F | F | F | F | F | F | F | F |
| 3 | AS | AS | 70,34 | -0,67 | NA | F | 2 | 0 | 0 | NA | 0 | 0 | F | F | F | F | F | F | F | F | F |
| 3 | AS | AS | 70,42 | -0,59 | NA | F | 3 | 0 | 0 | NA | 0 | 0 | F | F | F | F | F | F | F | F | F |
| 3 | AS | AS | 70,50 | -0,51 | NA | F | 4 | 0 | 0 | NA | 0 | 0 | F | F | F | F | F | F | F | F | F |
| 3 | AS | AS | 70,57 | -0,44 | NA | F | 5 | 0 | 0 | NA | 0 | 0 | F | F | F | F | F | F | F | F | F |
| 3 | AS | AS | 70,65 | -0,36 | NA | F | 6 | 0 | 0 | NA | 0 | 0 | F | F | F | F | F | F | F | F | F |
| 3 | AS | AS | 70,73 | -0,28 | NA | F | 7 | 0 | 0 | NA | 0 | 0 | F | F | F | F | F | F | F | F | F |
| 3 | AS | AS | 70,80 | -0,21 | NA | F | 8 | 0 | 0 | NA | 0 | 0 | F | F | F | F | F | F | F | F | F |
| 3 | AS | AS | 70,88 | -0,13 | NA | F | 9 | 0 | 0 | NA | 0 | 0 | F | F | F | F | F | F | F | F | F |
| 3 | AS | AS | 70,96 | -0,05 | NA | F | 10 | 0 | 0 | NA | 0 | 0 | F | F | F | F | F | F | F | F | F |
| 3 | RP | AS | 71,01 | 0,00 | NA | F | 0 | 0 | 0 | NA | 0 | 0 | F | F | F | T | F | F | F | F | F |
| 3 | RP | AS | 71,09 | 0,08 | NA | F | 1 | 0 | 0 | NA | 0 | 0 | F | F | F | F | T | F | F | F | F |
| 3 | RP | AS | 71,16 | 0,15 | NA | F | 2 | 0 | 0 | NA | 0 | 0 | F | F | F | F | T | F | F | F | F |
| 3 | RP | AS | 71,24 | 0,23 | NA | F | 3 | 0 | 0 | NA | 0 | 0 | F | F | F | F | F | T | F | F | F |
| 3 | RP | AS | 71,32 | 0,31 | NA | F | 4 | 0 | 0 | NA | 0 | 0 | F | F | F | F | F | T | F | F | F |
| 3 | RP | AS | 71,39 | 0,38 | NA | F | 5 | 0 | 0 | NA | 0 | 0 | F | F | F | F | F | T | F | F | F |
| 3 | RP | AS | 71,47 | 0,46 | NA | F | 6 | 0 | 0 | NA | 0 | 0 | F | F | F | F | F | F | F | F | F |
| 3 | RP | AS | 71,55 | 0,54 | NA | F | 7 | 0 | 0 | NA | 0 | 0 | F | F | F | F | F | F | F | F | F |
| 3 | RP | AS | 71,62 | 0,61 | NA | F | 8 | 0 | 0 | NA | 0 | 0 | F | F | F | F | F | F | F | F | F |
| 3 | RP | AS | 71,70 | 0,69 | NA | F | 9 | 0 | 0 | NA | 0 | 0 | F | F | F | F | F | F | F | F | F |
| 3 | GH | AS | 71,70 | 0,69 | NA | F | 0 | 0 | 0 | NA | 0 | 0 | F | F | F | F | F | F | F | F | F |
| 3 | GH | AS | 71,78 | 0,77 | NA | F | 1 | 0 | 0 | NA | 0 | 0 | F | F | F | F | F | F | F | F | F |
| 3 | GH | AS | 71,85 | 0,84 | NA | F | 2 | 0 | 0 | NA | 0 | 0 | F | F | F | F | F | F | F | F | F |
| 3 | GH | AS | 71,93 | 0,92 | NA | F | 3 | 0 | 0 | NA | 0 | 0 | F | F | F | F | F | F | F | F | F |
| 3 | GH | AS | 72,01 | 1,00 | NA | F | 4 | 0 | 0 | NA | 0 | 0 | F | F | F | F | F | F | F | F | F |
| 3 | GH | AS | 72,08 | 1,07 | NA | T | 5 | 0 | 0 | NA | 0 | 0 | F | F | F | F | F | F | F | F | F |

**Table A3**. R-codes used for subset selection and models 1, 2, 3a, and 3b.

| **Description** | **R-code** |
| --- | --- |
| Full data set | dd |
| Calculate outcome for Model 1 | dd$y.death <- dd$dead |
| Model 1: Vital status | mod.death <- glm(y. death ~ age * cci, family=binomial, data=dd) |
|  |  |
| Calculate if a CCI- change ≥ 1 has occurred | dd$y.any <- (dd$cci.change > 0) |
| Model 2: Any CCI change | mod.any <- glm(y.any ~ diag.state * age + tsc.1 + tsc.23 + tsc.46 + cci.fct + tsrp.1 + tsrp.23 + tsrp.46 + tsrt.1 + tsrt.23 + tsrt.46, family=binomial, data=dd) |
|  |  |
| Transform the CCI for Model 3a. | dd$y.pois <- ifelse(dd$cci.change < 6, dd$cci.change - 1, dd$cci.change - 6) |
| Select subset of data where a CCI-change took place | dd.change <- subset(dd.full, cci.change > 0) |
| Model 3a: Size of change using Poisson model | mod.pois <- glm(y.pois ~ diag.state * age + tsc.1 + tsc.23 + tsc.46 + cci.fct + tsrp.1 + tsrp.23 + tsrp.46 + tsrt.1 + tsrt.23 + tsrt.46, family=poisson, data=dd.change) |
|  |  |
| Calculate if a CCI- change ≥ 6 has occurred | dd$y.6p <- (dd$cci.change >= 6) |
| Mododel 3b: Size of change 6+ model. | mod.6p <- glm(y.6p ~ diag.state + age + tsc.1 + tsc.23 + tsc.46 + cci.fct.6p + cci, family=binomial, data=dd.change) |
